# Evaluation of EU air quality standards through modeling and the FAIRMODE benchmarking methodology

**DOI:** 10.1007/s11869-018-0631-z

**Published:** 2018-10-24

**Authors:** Jonilda Kushta, Georgios K. Georgiou, Yiannis Proestos, Theodoros Christoudias, Philippe Thunis, Chrysanthos Savvides, Christos Papadopoulos, Jos Lelieveld

**Affiliations:** 1 Energy, Environment and Water Research Centre (EEWRC), The Cyprus Institute, 20 Konstantinou Kavafi Street, Aglantzia, 2121 Nicosia, Cyprus; 2 Computation-based Science and Technology Research Centre (CaSToRC), The Cyprus Institute, 2121 Nicosia, Cyprus; 3 Joint Research Centre (JRC), Directorate for Energy, Transport and Climate, Air and Climate Unit, European Commission, Via E. Fermi 2749, I-21027 Ispra, VA Italy; 4 Department of Labour Inspection, Ministry of Labour, Welfare and Social Insurance, Nicosia, Cyprus; 5 Max Planck Institute for Chemistry, 55128 Mainz, Germany

**Keywords:** EU Air Quality Directive, Air pollution, Model evaluation, Cyprus, FAIRMODE, East Mediterranean

## Abstract

We evaluate air quality modeling over the East Mediterranean using the benchmarking methodology developed in the framework of the Forum for Air Quality Modelling in Europe (FAIRMODE). FAIRMODE aims to provide a harmonized approach of model evaluation for regulatory purposes. We test the methodology by assessing the performance of the Weather Research and Forecasting model coupled with chemistry (WRF-Chem) against ground-based air quality observations over Cyprus, a member state of the European Union. Two nested domains are used (at 50- and 10-km horizontal grid spacing) with the comparison performed over the innermost domain. We consider performance indicators reflecting regulations for air quality standards (maximum daily 8-hourly mean ozone, hourly nitrogen dioxide, and daily fine particulate matter concentrations). The WRF-Chem model is found to satisfy the proposed performance objectives regarding ozone and NO_2_, though it underestimates the latter in urban areas possibly due to uncertainties in emission inventories. Fine particulate matter is well represented by the model, except on days with strong influence from natural sources, highlighting the necessity for fine-tuning dust mobilization and transport in the region. The objectives are fulfilled even though discrepancies exist between model and observations. Our results indicate the need for more stringent performance criteria at relatively low concentrations. Overall, we find that the methodology provides in-depth information and relevant statistical metrics to guide air quality and model assessments for monitoring compliance with the EU Air Quality Directives and other guidelines to limit the impact of air pollution on human health and ecosystems.

**Electronic supplementary material:**

The online version of this article (10.1007/s11869-018-0631-z) contains supplementary material, which is available to authorized users.

## Introduction

Air quality is, and will likely continue to be, an important issue over the Eastern Mediterranean and the Middle East (EMME) region. Air pollution influences the quality of life, the number of premature deaths, and the frequency and severity of various respiratory diseases (Giannadaki et al. 2014; Lelieveld et al. 2014; Abdo et al. 2016; Khader et al. 2016; Dayan et al. 2017). The EMME region is a receptor of pollution from multiple sources of anthropogenic and natural origin (Lelieveld et al. 2002; Gerasopoulos et al. 2006; Mihalopoulos et al. 2007; Astitha and Kallos 2008; Im and Kanakidou 2012). The island of Cyprus, located centrally in the EMME region, is ideally located to assess current regulations and evaluate model-benchmarking methodologies. Cyprus is affected by long-range pollution transport from three continents (Europe, Africa, and Asia) and dust aerosols from the two largest desert regions in the world (North Africa and Middle East).

Station observations can only monitor the level of pollutants over specific locations, while models can provide insights into the emission, transport, and transformation of pollution over the whole region of interest. Therefore, models that exhibit satisfactory performance can complement observations or even replace them in cases of disoperation, for policy-related applications and reporting purposes. Thus, it is necessary to harmonize the criteria of model skill and capability to reproduce air quality features over a specific region, for officially reporting national air pollution levels and for examining compliance with regulations.

Model performance criteria are defined to benchmark a model application against agreed and/or regulated quality standards, by comparing statistical indicators against bound values. Specific validation protocols (i.e., Dennis et al. 2010 for assessment; Thunis and Clappier 2014 for emission scenarios) have been developed to support the use of air quality models when performing various tasks (assessment, forecasting, planning). A methodology for unified model evaluation process has been developed (Thunis et al. 2012a, b) in the framework of the Forum for Air Quality Modelling in Europe (FAIRMODE). The FAIRMODE model evaluation methodology aims at promoting and supporting the harmonized use of models by EU Member States, with emphasis on model application under the European Air Quality Directives. The approach is based on paired modeled and monitored data to offer diagnostics of model performance using various statistical indicators and diagrams. The FAIRMODE IDL-based DELTA software tool (http://aqm.jrc.ec.europa.eu/DELTA/) that incorporates this methodology can produce summary reports on performance indicators and guide model development to improve statistical metrics that are not in line with target values.

The FAIRMODE methodology has been used in the past to evaluate chemical transport models over Europe, by Carnevale et al. (2014) over the Po Valley and Georgieva et al. (2015) over Bulgaria. Recently, Monteiro et al. (2018) presented an analysis of the strengths and weaknesses of the FAIRMODE benchmarking approach, focusing on the pollutants regulated by the Air Quality Directive (PM2.5, NO_2_, and O_3_) and based on feedback from different research groups over Europe using continental, regional, and urban scale models. Through a Strengths, Weaknesses, Opportunities and Threats (SWOT) analysis, they identified the main advantages and value of the FAIRMODE approach compared to other methodologies. The main strengths are the successful promotion of harmonized reporting for air quality model applications to meet the relevant directives and the integration of the most essential (statistically and representatively) quality indicators. Weaknesses include among others the lack of a clear definition and use of measurement uncertainty for various pollutants, a significant component of the methodology. Evaluation and SWOT studies are essential and provide motivation and technical basis for a continuous improvement of the methodology. Evaluation and SWOT studies are essential and provide for motivation and technical basis for a continuous improvement of the methodology.

In this work, we apply the FAIRMODE methodology to evaluate the skill of the Weather Research and Forecasting model coupled with Chemistry (WRF-Chem) to simulate air quality for the year 2015. We have selected an integrated modeling system since the impact of aerosols on radiation, clouds, and precipitation remains an open research question, especially in our region of interest. Aerosols greatly influence the atmospheric processes over the EMME due to their abundance in the region from both natural (sea salt, mineral dust) and anthropogenic sources (Real and Sartelet 2011; Gerasopoulos et al. 2012; Kushta et al. 2014; Mailler et al. 2016; Abdelkader et al. 2017; Gkikas et al. 2018). Hence, models that incorporate the links and feedbacks between airborne pollution and atmospheric processes are needed (Grell and Baklanov 2011; Baklanov 2017).

The evaluation of WRF-Chem is based on the comparison of model results with available observations from the national air quality monitoring network. Ambient air quality is regulated in the EU member countries by the Directive 2008/50/EC. We model regulated pollutants, namely ozone (maximum daily 8-h mean O_3_), nitrogen dioxide (hourly NO_2_), and fine particulate matter (daily mean PM2.5 values), and discuss the use of model results in the context of the application of the EU Air Quality Directive for the country of Cyprus. We focus on the island of Cyprus to highlight strengths and weaknesses when applying such a methodology in countries of limited size with no land borders, subject to long-range transport of pollution from multiple sources, and with specific constrains in monitoring sites. Air quality over Cyprus is monitored through a network of 12 stations including five rural stations (four rural background and one industrial stations) and seven urban stations (four traffic and three urban background stations).

The paper is organized as follows: “Model and data” includes a description of the model data and observations used in this study, as well as the statistical background of the FAIRMODE evaluation methodology. In “Results and discussion,” we present the results of the model and we compare with observation for O_3_, NO_2_, and PM2.5. In “Concluding remarks,” we give our concluding remarks on the capabilities of the evaluation methodology and its use as a policy tool for air quality and pollutant-level exceedance reporting in European countries.

## Model and data

### Model description and input data

**Fig. 1:**
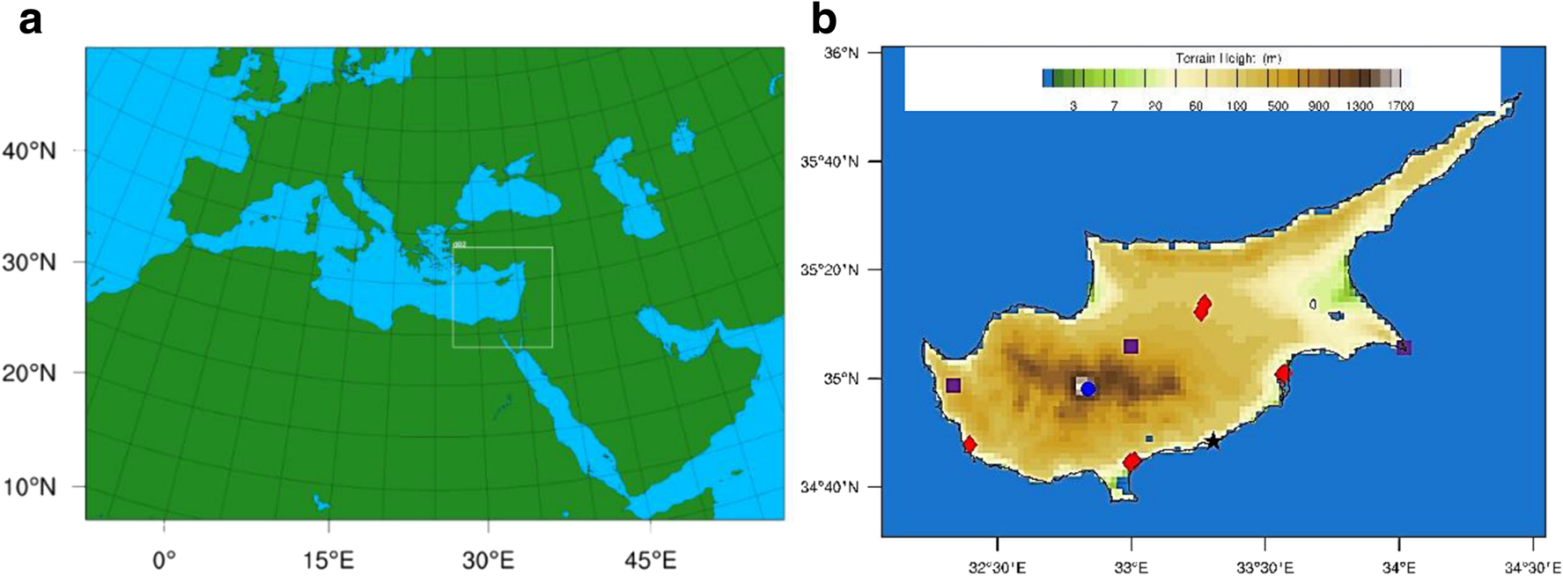
**a** The domains covered by this study: the parent domain covers Europe, North Africa, and the Arabian Peninsula at a horizontal resolution of 50 km, and the nested domain of the EMME has a grid spacing of 10 km. **b** The location and type of the air pollution monitoring stations over Cyprus. Rural background stations (3) are shown in purple squares, urban (background and traffic, 7 in total) in red, the Zygi industrial station as black star, and the Troodos rural background station, located above PBL, as blue circle

For this study, we use the coupled meteorological and atmospheric chemistry model WRF-Chem (v3.9.1.1) that simultaneously simulates physical and chemical processes taking into account direct and indirect feedbacks (Grell et al. 2005; Fast et al. 2006). The model is applied for a year-long period (2015) using two domains with respective grid spacing of 50 and 10 km with the nested one, covering the EMME countries, centered over Cyprus (Fig. 1a). The model physical and chemical parameterizations are summarized in Table 1. The parent domain has a large extent to cover important emission sources inside the regional model instead of representing them as lateral forcing from the global modeling system. WRF-Chem has been applied and evaluated for a summer period (July 2014), over Cyprus, in two recent studies by Kushta et al. (2017) and Georgiou et al. (2018). The model achieves an overall good representation of the unique geophysical features and atmospheric chemical composition of the region, considering the limitations imposed by uncertainties in the emission inventories and physical and chemical model parameterizations it is based on.

**Table 1:** Physical and chemical configuration of the modeling application

Process	Scheme/parameterization	Reference
Microphysics	Morrison 2-moment scheme	Morrison et al. 2005
Land surface	NOAH land surface model	Chen and Dudhia 2001
Boundary layer	Yonsei University (YSU) planetary boundary layer	Hong et al. 2006
Cumulus	Grell 3D ensemble scheme	Grell and Devenyi 2002
Surface layer	MM5 similarity surface layer scheme	Zhang and Anthes 1982
Radiation	Rapid Radiative Transfer Model (RRTTM)	Iacono et al. 2008
Gas-phase chemistry	RACM regional atmospheric chemistry mechanism	Stockwell et al. 1997
Aerosols	Modal Aerosol Dynamics Model for Europe (MADE), Secondary Organic Aerosol Model (SORGAM)	Ackermann et al. 1998, Schell et al. 2001

The WRF-Chem model meteorology is driven by 6-hourly boundary conditions at 0.5° × 0.5° horizontal resolution from the US National Centers for Environmental Prediction (NCEP) global forecast system (GFS). The modeled chemistry uses boundary conditions from the global Model for Ozone And Related chemical Tracers (MOZART version 4) for the outermost domain (Emmons et al. 2010). The nested domain receives its meteorological and chemical lateral conditions from the coarse domain. The emissions of natural aerosol species (mineral dust, sea salt) are simulated online using the parameterization schemes included in the model. Biogenic emissions of volatile organic species are derived from the Model on Emissions of Gases and Aerosols from Nature (MEGAN), as described in Guenther et al. (2006).

The anthropogenic emissions are based on the EDGAR-HTAP v2 emission estimates for 2010 at a resolution of 0.1° × 0.1°, with no further modifications to incorporate changes that may have occurred in the recent years in the region (Janssens-Maenhout et al. 2012). The emission inventory is processed for spatial and temporal allocation to the resolution of the model domains and lumped speciation to match the species of the chemical mechanisms used (Kushta et al. 2017).

### Observations

The model performance is evaluated against hourly measurements of the national monitoring network for the year 2015 over Cyprus. The Cypriot Legal Framework on Ambient Air Quality comprises the Quality of Ambient Air Law of 2010 (Ν. 77(Ι)/2010) and two series of Regulations that determine limits for the concentrations of certain pollutants in ambient air, in line with the European regulations on ambient air quality. The implementation and management of the law and regulations are the responsibility of the Ministry of Labour and Social Insurance Department of Labour Inspection (DLI). The DLI air quality monitoring network consists of four rural background stations (one of which located above the atmospheric boundary layer in the Troodos mountain), seven urban stations comprising of three urban background (hereafter referred to as urban) and four urban traffic stations (traffic), and a station located in the vicinity of the coastal, heavy-industry zone of Zygi (industrial). Station classification information, following the Exchange of Information Decision (EOI 97/101/EC 1997) and the Implementation Decision for Reporting (2011/850/EU 2011), and the availability of measurements per station and per species are summarized in Table 2. We focus on the atmospheric pollutants included in the air quality directives and supported by the evaluation software: hourly values regarding NO_2_, mean daily values for PM2.5, and daily maximum 8-h O_3_. The analysis is performed in volume mixing ratio units (ppbV) for gaseous pollutants and in mass concentration units (μg m^−3^) for particulate matter that are directly comparable as they are the original units for both observations and model results.

**Table 2:** The air quality monitoring network of Cyprus (station, type, availability of observations for the respective pollutant)

Station	Type of area	Type of station	NO	NO_2_	SO_2_	O_3_	CO	PM10	PM2.5	Benzene
Agia Marina	Rural	Background	X	X	X	X	X	X	X	X
Cavo Greco	Rural	Background	X	X		X				
Inia	Rural	Background	X	X		X				
Troodos	Rural	Background	X	X		X				
Larnaca Res	Urban	Background	X	X	X	X	X			
Larnaca Tra	Urban	Traffic	X	X	X	X	X	X	X	X
Limassol Res	Urban	Background	X	X	X	X	X			
Limassol Tra	Urban	Traffic	X	X	X	X	X	X		X
Nicosia Res	Urban	Background	X	X	X	X	X			X
Nicosia Tra	Urban	Traffic	X	X	X	X	X	X	X	X
Pafos	Urban	Traffic	X	X	X	X	X	X		X
Zygi	Rural	Industrial	X	X	X	X		X	X	

As shown in Fig. 1b, the rural background stations are located along the central west-east axis of the island monitoring air pollution transport over the country under the main atmospheric circulation regimes (westerlies and easterlies). The coastal stations capture the impact of local emissions and/or long-range transport of pollutants depending on the dominating wind direction (Tyrlis and Lelieveld 2012; Tyrlis et al. 2014). The rural background stations are all used in the analysis regarding ozone and nitrogen dioxide. The urban background and traffic stations are located within the major urban areas (Nicosia, Limassol, Larnaca, and Pafos). For our study, we only use the urban background stations for the gaseous pollutants since the traffic stations capture high spikes in local emissions (especially NOx) due to their proximity to main traffic routes that cannot be resolved at the horizontal resolution of a regional-scale air quality model (10–50 km). Regarding fine particulate matter, only one rural background station has available PM2.5 observations (Agia Marina). There are no other stations located in rural areas that can contribute to the present PM2.5 analysis. The urban traffic stations provide additional measurements of particulate matter; thus, we include these stations in our analysis of aerosol pollutants, to investigate the performance criteria stringency, even though the methodology is not designed for application in traffic stations.

### Benchmarking evaluation methodology

#### Modeling quality indicator

The overall benchmarking evaluation procedure used in this study is presented in detail in Thunis et al. (2012a, b). The methodology works on paired series of model and observed values of the respective pollutant for year-long periods. It investigates the model capabilities by introducing an overall indicator, namely, the modeling quality indicator (MQI), taking into account the measurement uncertainty of each pollutant. The measurement uncertainty parameters currently used in the FAIRMODE methodology are discussed in detail in Thunis et al. (2013) for ozone and in Pernigotti et al. (2013) for coarse particulate matter and nitrogen dioxide.

MQI defines the deviation between measured (O) and modeled (M) values at a given *i* time (hour or day) as a factor of the measurement uncertainty and a scaling factor that indicates the stringency of the objective to be satisfied (e.g., a scaling factor of 2 means the allowed deviation should be within a factor of 2 of the measurement uncertainty gap):

$$ MQI=\frac{\mid Oi- Mi\mid }{\beta\ {U}_{95}\ (Oi)},\beta =2 $$

*O*_i_ and *M*_i_ are the observed and modeled values, respectively, *U*_95_ is the 95th percentile measurement uncertainty of the observed concentration level, and *β* is the coefficient that scales the proportionality of the bias to the measurement uncertainty (Thunis et al. 2012b). MQI can be generalized to a yearly time series as follows:

$$ MQI=\frac{RMSE}{\beta\ RMSu} $$

*where RMSu* is the root mean square of the measurement uncertainty (details in Thunis et al. 2013; Pernigotti et al. 2013) and RMSE is the root mean square error between model and measured concentrations. For annual values, *O*_i_ and *M*_i_ are substituted in the formula by the observed and modeled annual mean, respectively, and the uncertainty is calculated at the 95th percentile upon the annual mean measured concentration. The modeling quality objective (MQO) is the criterion for the value of the MQI to be satisfied for satisfactory model performance in terms of air quality representation for reporting applications (minimum level of quality). MQO is fulfilled if the MQI is less than or equal to unity for at least 90% of the available monitoring data.

The measurement uncertainty parameter is a significant component of the evaluation methodology reflecting our confidence level in the observational data. Initially, the measurement uncertainty of the benchmarking methodology was set to be constant (independent of the concentration level of the respective pollutant) and defined by the data quality objective (DQO) value of the Air Quality Directive to 15, 15, and 25% for O_3_, NO_2_, and PM10, respectively (Thunis et al. 2012a, b). Thunis et al. (2013) reshaped the measurement uncertainty as a combination of two components, one given as a function of the pollutant concentration and a non-proportional component (independent of measured values) calculated on a concentration level of choice (reference value, RV). The total measurement uncertainty represents the 95th percentile highest value among all uncertainty values calculated for each pollutant. The calculations are performed on data from JRC instrument inter-comparison results (Lagler et al. 2011) for particulate matter, EU AIRBASE stations for series of meteorological years for NO_2_, and analytical relationships for O_3_. Several updates of the uncertainty parameters used to estimate the measurement uncertainty for particulate matter have been made (see “Guidance document on modeling quality objectives and benchmarking” (FAIRMODE 2017) with current values reflecting uncertainties associated to different types of instrumentations (e.g., β-ray measurement technique).

For the visualization of the MQO, a target diagram adapted from Jolliff et al. (2009) is used (results of current study shown in Fig. 3, top plots). The horizontal axis represents the central root mean square error (CRMSE) while the vertical axis refers to BIAS, normalized by measurement uncertainty of the respective pollutant levels. For each station, a dot is placed on the target diagram and the distance of the dot from the center of the diagram represents the MQI for that station. Stations with MQI within the green area are identified as stations that fulfill the performance criteria. The dashed line represents the limit outside which (but still within the green area) model results are within the measurement uncertainty range. When the MQI of a specific station is greater than one and falls out of the colored area, there are statistically significant differences between model and observations. Four zones on the plot help the user identify the reasons for model-observation differences in terms of standard deviation (SD), bias, and correlation (*R*) (Thunis et al. 2012a, b; Jolliff et al. 2009). The top (bottom) two zones exhibit positive (negative) bias while the assignment of the station to the left (right) zone of the graph indicates a unsystematic (systematic) RMSE ratio (error dominated by SD or by *R*). Model performance indicators through the “target” approach have been assessed in Pederzoli et al. (2012).

#### Model performance indicators

The MQI proposed in the framework of the FAIRMODE benchmarking methodology combines the bias error (BIAS), standard deviation (σ), and correlation (R) into a single number, providing a general measure of the model performance associated with RMSE. These three statistical metrics are related as follows:

$$ {MQI}^2=\frac{RMSE^2}{{\left(\beta\ RMSu\right)}^2}=\frac{BIAS^2}{{\left(\beta\ RMSu\right)}^2}+\frac{{\left({\sigma}_M-{\sigma}_{\mathrm{o}}\right)}^2}{{\left(\beta\ RMSu\right)}^2}+\frac{2{\sigma}_{\mathrm{o}}{\sigma}_{\mathrm{M}}\left(1-R\right)}{{\left(\beta\ RMSu\right)}^2} $$

Assuming ideal cases where two out of the three statistical indicators perform perfectly, we get model performance indicators (MPI) that depend solely on the third remaining metric. MPIs associated separately with correlation, standard deviation, and bias can then be used to highlight which aspect of the model performance can be improved. Model performance criteria (MPC) are defined for each MPI to be fulfilled during model evaluation.

That is, assuming bias to be zero and the standard deviation of observations equal to that of modeled values leads to the following condition on *R*:

$$ R>1-0.5\ {\beta}^2\frac{RMSu^2}{\sigma_{\mathrm{o}}{\sigma}_{\mathrm{M}}} $$

where *σ*_o_ and *σ*_M_ are the standard deviation of the observed and modeled concentrations, respectively. Similarly, the MPIs for bias and standard deviation translate in the following conditions:

$$ \kern2.75em \left| BIAS\right|\le \beta RMSu $$

$$ \kern2.25em \left|{\sigma}_{\mathrm{M}}-{\sigma}_{\mathrm{o}}\right|\le \beta RMSu $$

It is important to note that the model performance criteria for bias, correlation, and standard deviation represent necessary but not sufficient conditions to ensure fulfillment of the MQO.

## Results and discussion

We focus on the pollutant metrics as required by policy applications: the maximum daily 8-hourly mean O_3_, hourly NO_2_, and mean daily PM2.5 concentrations. The tool additionally enables the investigation of other metrics (hourly concentrations for all pollutants). We have not performed an analysis on the coarse particulate matter (PM10) in the current study. The fraction of particles with diameter between 2.5 and 10 μm in the region is dominated by natural aerosols (mineral dust) that can introduce large uncertainties to the evaluation procedure without a separate assessment. As shown in Karanasiou et al. (2009) and Chalbot et al. (2013), crustal soil particles typically concentrate into the coarse fraction mode of particulate matter and only a small fraction of them (5–10%) are present in the fine mode (diameter less than 2.5 μm).

All the stations included in the analysis satisfy the 75% data availability threshold, as required by the methodology. The EU Air Quality Policy introduced as Air Quality Standards by the Directive 2008/50/EU for the Member States defines a maximum threshold of 25 (current) and 20 μg m^−3^ (future) for mean annual PM2.5 concentration, 120 μg m^−3^ for maximum daily 8-h mean O_3_ not to be exceeded over 25 days (averaged over a 3-year period), and 40 (200) μg m^−3^ for annual (hourly) NO_2_ concentrations. The target plot analysis and threshold exceedance assessment of the gaseous pollutants are performed separately on the urban and rural background stations as they exhibit dissimilar characteristics. The main statistical indicators (bar plots of mean, standard deviation, bias, etc.) include both categories of stations (rural and urban) for an overview of the model performance.

### Ozone

The bar plots of mean annual values of the daily max 8-h O_3_ concentrations (Online Resource, Fig. S1a) indicate that the model is capable of reproducing the distribution of ozone over the island. An overestimation is noticeable in urban areas. This overestimation may be related to the lateral forcing from the global model and low local emission fluxes of ozone precursors such as nitrogen oxides, as shown in Kushta et al. (2017). There is a difference of daily max 8-h O_3_ values between rural and urban background stations that leads to lower annual mean max 8-h concentrations in the urban sites as a result of ozone titration by nitric oxide from traffic and residential activities. This outcome highlights a local-scale intense conversion effect (of O_3_ by NO into NO_2_) near emission sources where higher levels of NOx emissions are present relative to rural areas. The standard deviation ranges from 6.5 to 15.2 ppbV, with areas under “clean air” influence, like the Inia station located in the westernmost part of the island (affected by the prevailing north–northwest wind flow) and the Troodos station (mountainous site above boundary layer) exhibiting the largest variations in max 8-h O_3_ levels (Online Resource, Fig. S1b). The model shows a smoother variation in terms of standard deviation of about 9 ± 2 ppbV. The mean bias and correlation coefficients are larger over the urban stations (Online Resource, Figs. S1c and S1d). While the model cannot reproduce the magnitude of ozone in urban background sites (large mean bias), the variation correlates better due to the more pronounced photochemical processes. In rural background stations, the mean bias is lower (modeled values are closer to the observed ones) but the correlations lack accuracy mostly due to the influence of long-range transported pollution with no distinguishable temporal variation in the production and depletion life cycle. The fact that the Troodos station (not influenced by the boundary layer) exhibits smaller mean bias despite its large observed standard deviation underlines the significance of accurately representing the boundary layer in the simulation of pollutant advection and diffusion.

**Fig. 2:**
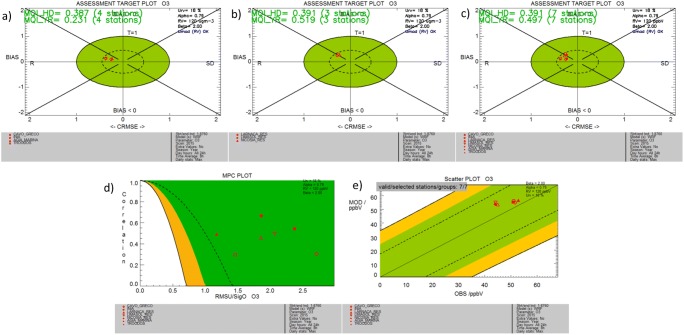
Assessment target plots (top row) for **a** rural background, **b** urban background, and **c** all stations. **d** Model performance criteria (bottom row) associated with correlation coefficient and **e** scatter plot of mean annual values of 8-h max ozone

We next examine the compliance with the model performance objectives as defined by the evaluation methodology. Figure 2 (top plots) presents the assessment target plot of the MQI for rural background (Fig. 2a), urban background stations (Fig. 2b), and all stations combined (Fig. 2c). The model quality objective requires a MQI of less than unity for at least 90% of the stations included in the analysis. As depicted on the top panel plots, the MQI for both group of stations (four background and three residential stations) is satisfied with MQI_rural_ = 0.387 and MQI_urban_ = 0.391 for hourly values and MQI_rural_ = 0.231 and MQI_urban_ = 0.519 for annual means under a measurement uncertainty of 18%. For all stations combined, the MQI_all_ is 0.4 for the hourly assessment and 0.5 for the annual mean values, again satisfying the MQO of less than one (Fig. 2c). A small positive bias of the model is evident due to the location of the station dots in the upper part of the target diagram. The statistical metric of correlation is the one with less satisfactory performance.

The model performance criteria related to the MPI for correlation is given in Fig. 2d for all types of stations (rural and urban background sites). All stations satisfy the MPI for correlation (as well as the MPI for standard deviation and mean bias, not shown). There is a clear distinction between observed mean annual values for averaged max 8-h O_3_ concentrations in rural versus urban background stations (Fig. 2e), but they remain close in their respective modeled values (~ 50 ppbV).

**Fig. 3:**
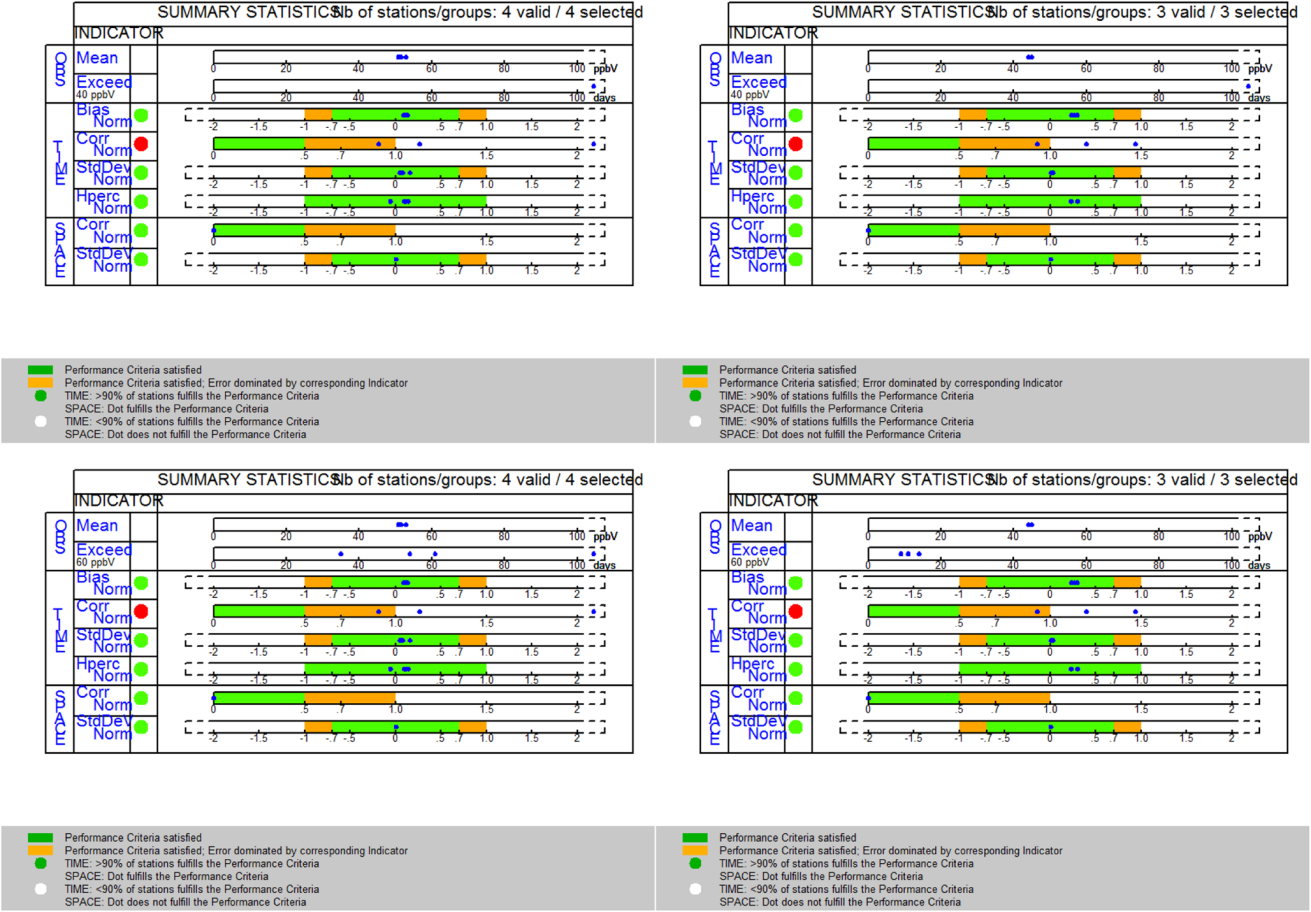
Summary report of threshold exceedances (with threshold concentrations set to 40 ppbV for top row and 60 ppbV for bottom row) and model performance statistical metrics of 8-h max ozone for the rural background stations on the left column (Cavo Greco, Inia, Agia Marina, and Troodos) and urban background stations on the right column (Larnaca, Limassol, and Nicosia)

Another component of the benchmarking methodology is the assessment of threshold exceedances and the provision of a summary report that includes information on the model performance (temporal and spatial statistical indicators) and exceedances for all stations in one diagram. As seen in Fig. 3, for Cyprus in 2015, the mean max 8-h O_3_ over the rural background stations is approximately 50–53 ppbV while in the urban background stations, it is almost constant in all cities at about 45 ppbV (row 1 of summary statistics plot in Fig. 3). Row 2 shows the number of days with exceedances of the threshold set by the user. We performed a threshold analysis for two values, 40 ppbV and 60 ppbV, to account for the impact of ozone on both vegetation and human health, respectively. The value of 40 ppbV has been proposed in the second edition of the World Health Organization Air Quality Guidelines for Europe (WHO 2000). The assessment of threshold exceedances over urban areas is important for human health; the analysis of the respective situation in vegetated areas is of equal importance for ecosystems.

The rural background concentrations of ozone over Cyprus are consistently 5–10 ppbV higher than urban levels. From the summary plot (Fig. 3, second row), we can see that in all rural background stations (important for vegetation and agricultural crops), the threshold of 40 ppbV is exceeded at all stations for more than 100 days. In urban background sites, where population density is larger, there occur 10–15 days when the 60-ppbV threshold is exceeded that are within the 25 exceedance days limit set by the EU. We note that the EU directive refers to the average—over 3 years—value; therefore, a continuous longer-term assessment should be performed for regulatory purposes.

Regarding model performance, rows 3 to 6 of the summary plot provide an overview of the temporal statistics for bias, correlation, standard deviation and a metric of the ability of the model to capture the highest range of concentration values (the latter still under development). Rows 7 and 8, in turn, provide metrics of the spatial performance of the model in terms of correlation and standard deviation. Average values for each station (over the selected time period) are calculated, and subsequently, the spatial correlation and standard deviation are computed. All indicators are normalized by the measurement uncertainty. In each statistical metric (row), the fulfillment of the performance criteria is shown with the green-shaded area. The orange area depicts regions where the criteria are fulfilled but model performance is dominated by the respective error. As shown in Fig. 3, the temporal and spatial statistical indicators are satisfied for both rural and urban background stations, for all metrics (bias, correlation, and standard deviation in time; correlation and standard deviation in space). The red dot in the temporal correlation MPI indicates that the time correlation–associated error is the one that dominates the model performance quality.

### Nitrogen dioxide

A similar analysis is performed for hourly nitrogen dioxide concentrations showing an overall fulfillment of the objectives set by the methodology. The mean, standard deviation, mean bias, and correlation reveal that there is a significant underestimation of the mean concentrations and variation of NO_2_ over the urban background stations with respective modeled values being 50% less than observed (Online Resource, Fig. S2). The mean bias is low in the rural background stations; in urban areas, where the horizontal resolution of the regional model (10 km) cannot accurately reproduce the magnitude and variation of the local emissions, the bias is mostly negative between − 5 and − 6 ppbV. The correlation of the observed and modeled hourly values varies from 0.2 in rural background stations to 0.6 in urban background stations. The low correlation in rural stations is an outcome of the low and relatively constant NO_2_ concentrations in these areas, with no distinct diurnal cycle and background intensity. In urban areas, the correlations are higher but still dominated by possible discrepancies in the spatial and temporal evolution of emission fluxes related to residential activities. Overall, the NO_2_ levels over Cyprus are relatively low and well within the regulation limits.

**Fig. 4:**
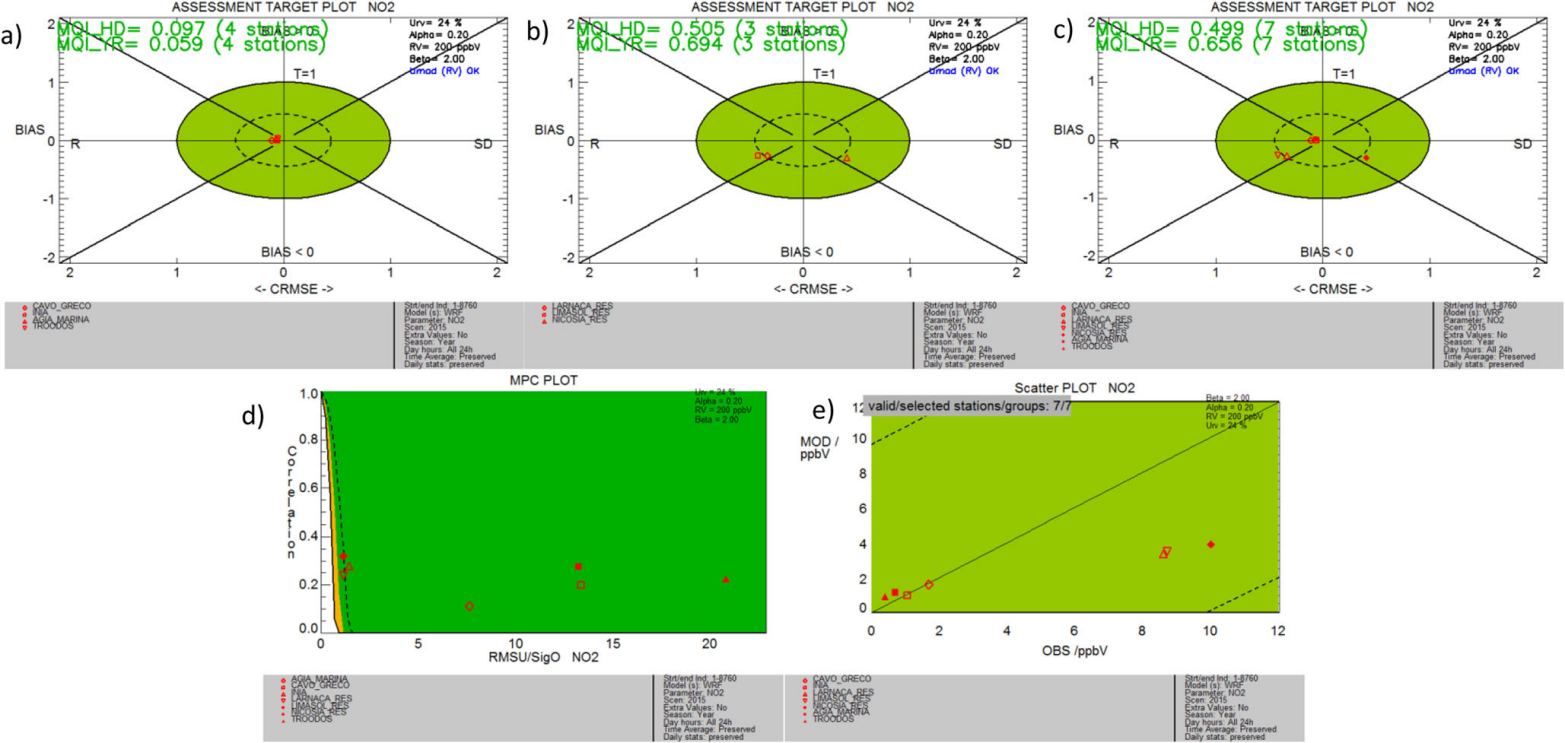
As in Fig. 2 but for hourly NO_2_

The MQI are satisfied in both rural and urban stations as shown in Fig. 4. At the rural background stations (Fig. 4a), the objectives are met more closely with both annual and hourly MQI less than 0.1. At the urban stations (Fig. 4b), the MQI is again below unity with the hourly MQI = 0.5 and the yearly MQI = 0.7. The overall MQIs of all stations combined are closer to the values of the urban stations (0.5 and 0.66 for hourly and annual data, respectively). The two coastal stations (Larnaca and Limassol) are located on the left side of the plot indicating that correlation is the lowest performing metric, while Nicosia is on the right part indicating that standard deviation is captured poorly. Despite the low correlation, the model performance criteria for all stations are within the green area in the MPC plots (Fig. 4d) with smaller RMSu to *σ*_o_ ratio at urban stations as expected, mostly due to the low NO_2_ values over the island. There are no hourly or annual threshold exceedances at any of the stations for 2015. Figure 4e shows the large variation in observed nitrogen dioxide levels varying between ~ 1 ppbV for the rural background stations (well reproduced by the model) and ~ 8–10 ppbV over urban areas (strongly underestimated by the model with a value of ~ 2–4 ppbV). Meeting the target set by the methodology, despite the evident discrepancies between modeled and observed values in the urban stations, is a result of the low NO_2_ levels at these stations. Low concentrations are indeed associated to large measurement uncertainties. In this methodology, the performance criteria are strongly linked to the uncertainty gap, meaning that low concentrations with large measurement uncertainty lead to easily satisfied criteria. For policy applications, however, there is no need for a more rigorous assessment since annual and hourly concentrations are below the threshold set by the air quality directive.

The underestimation in NO_2_ levels may result from limitations in the resolution of national emissions in these areas as represented in the emission database (~ 10-km resolution, same as the nested grid spacing). Several other studies have highlighted the discrepancies between different emission inventories, obtained with similar methodology and resolution (top-down regional or global inventories) or with different scales and approaches. Lopez et al. (2017) compared fine scale bottom-up urban emission inventories with regional top-down datasets and found large discrepancies in NOx and fine and coarse particulate matter, both in totals and sectorial emissions, with the regional emission inventories underestimating by 20–80% the NOx and 50–90% the PM10 emission fluxes. The authors indicated that the activity data based on fuel sales and population, used in the compilation of the regional inventories, versus actual traffic volume used in the bottom-up inventories, is the dominating reason for the underestimation of NO_2_ emissions. Moreover, even regional emission inventories based on top-down approaches exhibit substantial differences over urban areas, especially for NOx and VOCs, in terms of total, sectorial emissions, and spatial distribution, possibly due to downscaling approaches and choice of spatial proxies (Trombetti et al. 2018). Thus, the accurate modeling of NOx fluxes at the urban scale requires higher resolution in both emission data and simulations and preferably bottom-up emission inventories.

Specifically for the country of Cyprus, studies indicate that the global top-down anthropogenic inventories do not adequately represent the gradient of emission fluxes between rural and populated areas, leading to an underestimation in modeled nitrogen oxide levels and overestimation of ozone levels in urban zones (Kushta et al. 2017; Georgiou et al. 2018). It is anticipated that the use of an up-to-date high-resolution (< 2 km) and temporally resolved (diurnal to seasonal) national emission inventory encompassing ground-based and satellite information and an accompanying model configuration with similar grid spacing can help capture the magnitude of emission fluxes and ozone levels in urban areas.

### Fine particulate matter

The fine particulate matter (PM2.5) analysis is performed using daily mean values. Cyprus is frequently affected by dust storms from both North Africa and the Middle East (Kallos et al. 2014; Gkikas et al. 2018; Solomos et al. 2017, 2018), and these events can contribute to the overall exceedances of the health safety thresholds set by the Air Quality Directive. The WRF-Chem model includes an online dust mobilization and transport module that simulates the sources and transport of mineral dust particles in the atmosphere. We note that an evaluation and fine tuning of its performance for the region of this study have not been performed in an integrated way, other than event-based analyses. The situation in the Eastern Mediterranean is complex due the proximity (and influence) of two large dust sources, North Africa and the Middle East.

During September 2015, a large dust event over the EMME region originated in Syria through the inert forcing of a thermal low developed in the area and strengthened by the convective activity over northern Iraq (haboob forming mechanism) that merged with and enhanced the dust cloud mobilization and transport (Gasch et al. 2017; Solomos et al. 2017). The study of Solomos et al. (2017) showed that the default land use–type dataset used by the model (USGS Global Land Cover Characteristics Data Base Version 2) does not include very active dust sources of uncultivated fields (especially around the Euphrates River) that are a result of a disruption in summer agriculture activities either due to climatic forcing in the region and/or, mainly, military conflict. These changes in land use due to war in the areas of northern Iraq and Syria significantly affect model performance. Additionally, relatively higher resolution (finer than 4 km over dust sources) is needed to accurately simulate convective processes and vertical motion for dust uplifting. Over Cyprus, the study also showed that the model cannot reproduce the magnitude of the observed fine and coarse particulate matter concentrations measured at the coastal and inland stations, as well as ground-based LIDAR vertical distributions. The authors attributed the discrepancies to several dust and atmospheric processes such as an underestimation of the intense downward mixing and emission fluxes at the source area, model limitations due to the size distribution and deposition rates. Thus, to avoid misleading results regarding the model performance over the rest of the year, we exclude the 2 days of the dust episode from our analysis (by omitting only PM2.5 concentrations above 100 μg m^−3^ on 8 and 9 September). This is the only exclusion performed on the observational data on a year during which other dust episodes also occurred.

For the PM2.5 assessment, we include traffic stations in the analysis since aerosol measurements are not available at urban background stations. We also include measurements performed at the Zygi station despite its classification as an industrial site. These decisions are driven by the scarcity of measurements. We however need to remember these choices when analyzing the results. From the rural background stations, only Agia Marina has a set of complete observations for the year 2015.

The model captures the magnitude and standard deviation of the fine particulate matter in the area with a slight overestimation over Agia Marina (rural background) and an underestimation of urban levels by 2–3 μg m^−3^ (Online Resource, Fig. S3). Modeled yearly averaged PM2.5 concentrations are above the 10-μg m^−3^ threshold of the World Health Organization (WHO) for all four stations, but within the annual threshold of 25 (current) and 20 μg m^−3^ (future) as defined in the Air Quality Standards by the Directive 2008/50/EU. The observed annual mean at Agia Marina is below this value indicating that the model shows a non-measured annual exceedance. Exceedances at the other stations are adequately simulated by the model. The largest biases (negative in sign) and the largest correlations are over the urban stations of Larnaca and Nicosia (Online Resource, Fig. S3c, d).

**Fig. 5:**
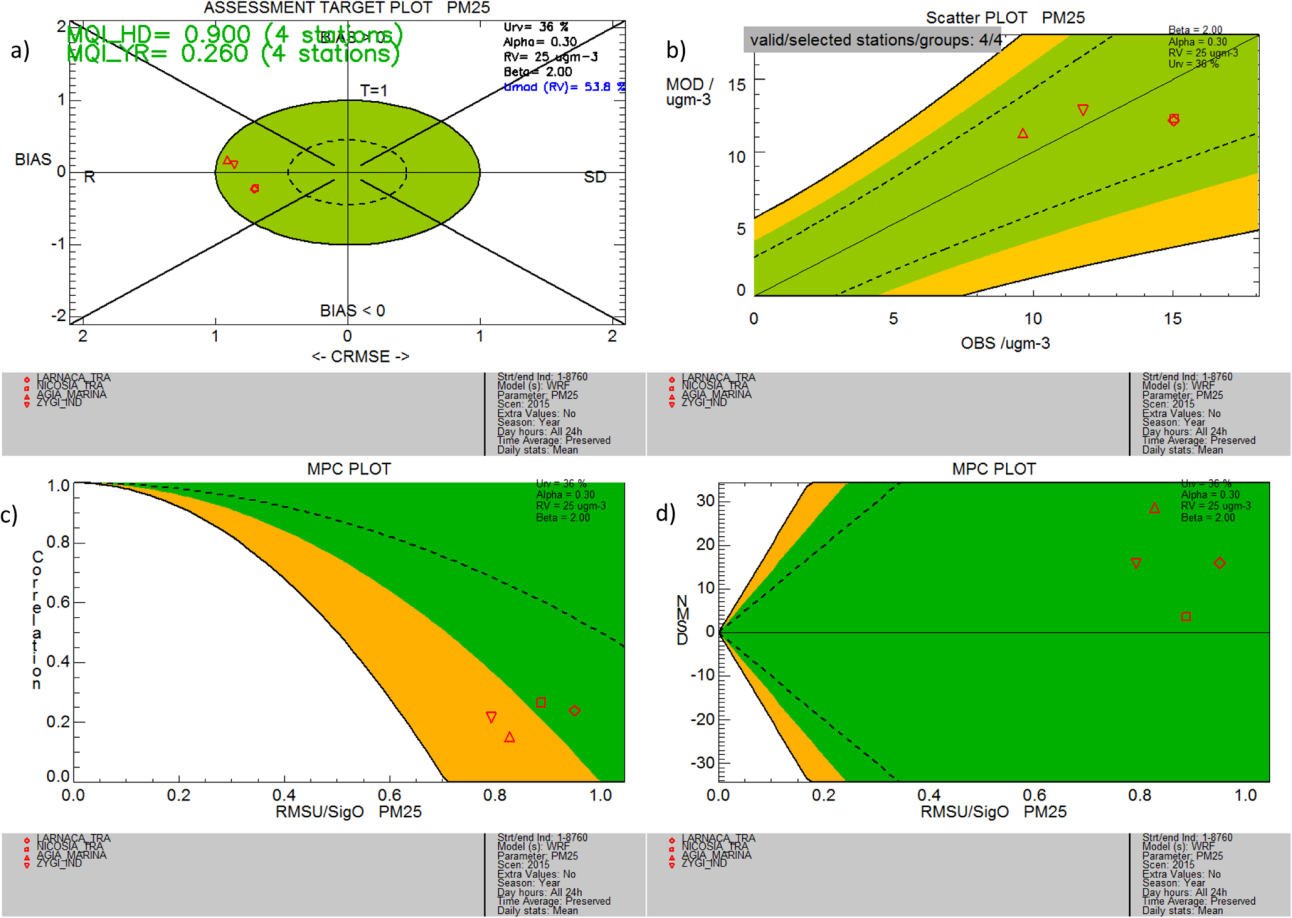
**a** Target plot and **b** scatter plot of mean annual PM2.5 concentrations and model performance criteria for **c** correlation and **d** standard deviation calculated on mean daily PM2.5 values for two urban background stations (Nicosia and Larnaca), one rural background station (Agia Marina), and one industrial station (Zygi)

The model fulfills the performance criteria for normalized standard deviation and correlation coefficient (Fig. 5a). The error related to the correlation coefficient dominates the model performance for both background and industrial stations. This is also shown in the MPC plots (Fig. 5, bottom plots) with the MPC for correlation in the orange area (Agia Marina and Zygi). The scatter plot of the mean annual values reveals a smaller range of model variation of PM2.5 distribution from site to site than in the observations (Fig. 5b). All model results fall within the green target area and significantly close to the 1:1 line. We note that even though the model evaluation benchmarking methodology is not fit for traffic and industrial sites, the model objectives are also satisfied over these sites. As seen in Online Resource, Fig. S3, there is no common pattern in the PM2.5 pollution characteristics of the traffic, industrial, and rural background stations. Therefore, the objective fulfillment highlights the need to scrutinize whether the MQO are stringent enough regarding daily fine particulate matter.

**Fig. 6:**
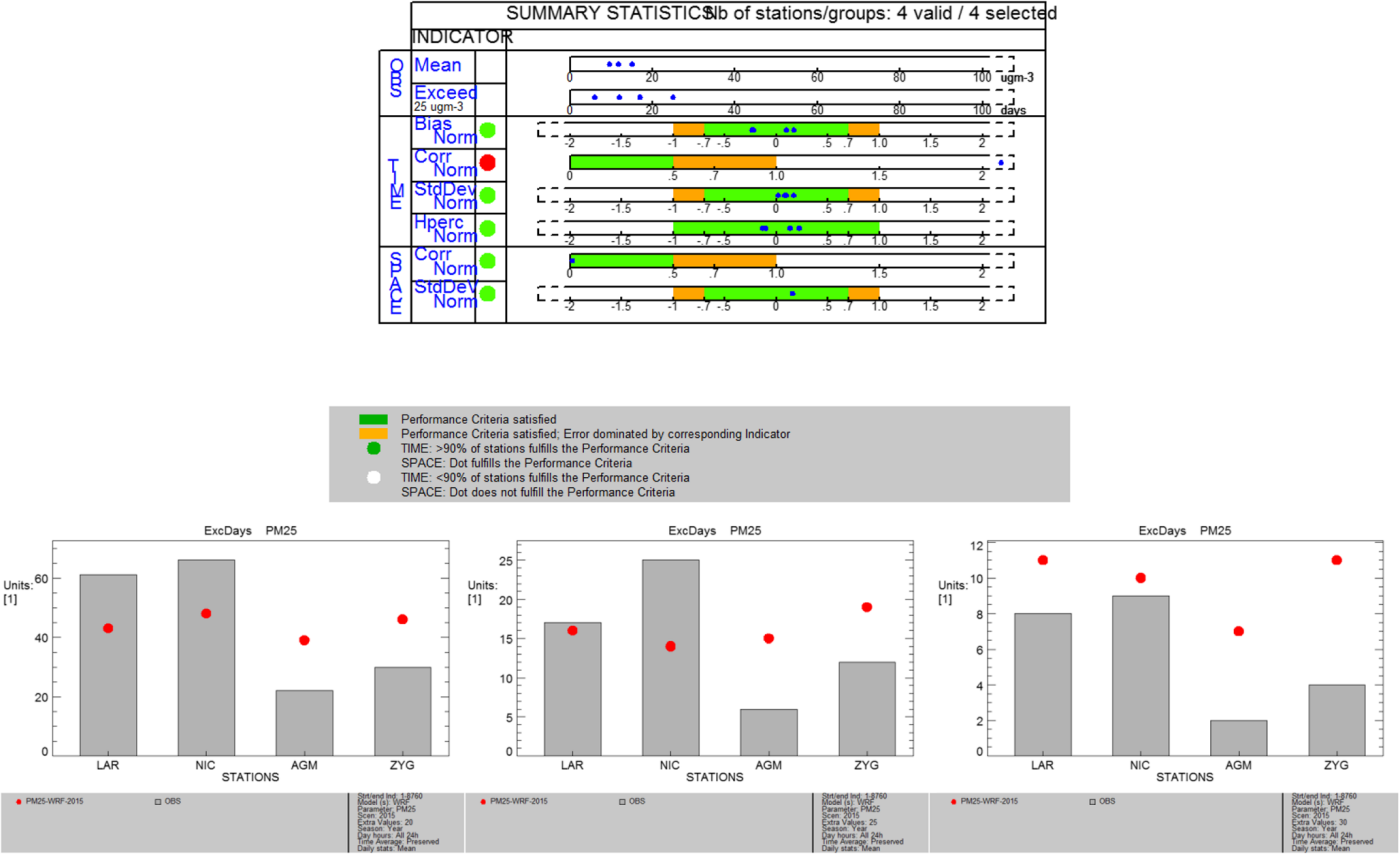
**a**–**d** Summary report (top panel) and threshold exceedance plot for 20 μg m^−3^ (left bottom plot), 25 μg m^−3^ (middle bottom plot), and 30 μg m^−3^ (right bottom plot) for mean daily PM2.5 concentrations for two urban background stations (Nicosia and Larnaca), one rural background station (Agia Marina), and one industrial station (Zygi)

We also present the summary report and threshold exceedance analysis for PM2.5 (Fig. 6). There is no threshold value in the Air Quality Directive regarding mean daily PM2.5 exceedance days (row 2). We use the values of 20, 25, and 30 μg m^−3^ to assess the consistency of the methodology (the summary plot shown in Fig. 6a refers to 25 μg m^−3^). The thresholds set by the National Ambient Air Quality Standards (NAAQS) for the USA (65 μg m^−3^) and SEPA, Vehicle Emission Control Center for China in residential areas (50 μg m^−3^), are only exceeded for 1 and 2 days, respectively, during the dust event described above. As shown in the threshold exceedance plots (Fig. 6b for 20, Fig. 6c for 25, and Fig. 6d for 30 μg m^−3^), the model misses a number of exceedance days in the urban sites (Nicosia and Larnaca), overestimates the threshold exceedances in the rural background station of Agia Marina and the industrial site of Zygi for the thresholds below 25 μg m^−3^, and overestimates exceedance days in all stations for the upper threshold of 30 μg m^−3^. These results indicate a need for a more detailed investigation of the sources of pollution during days with high PM2.5 concentrations and the influence of natural versus anthropogenic pollution. It is anticipated that local emission information at higher resolution as well as a dedicated analysis of the natural aerosol sources in the region and their representation in the model will contribute to the improvement of the model performance regarding particulate matter. This is a necessary step towards creating methodologies to assess the contribution of such sources for policy-related applications.

## Concluding remarks

We performed a statistical analysis of the regional atmospheric and chemistry model WRF-Chem using the benchmarking evaluation methodology developed in the framework of FAIRMODE, driven by the necessity of harmonizing model evaluation criteria for regulatory purposes. We applied the evaluation procedure over Cyprus, an EU member country located centrally in the Eastern Mediterranean and Middle East region, being an ideal case study due to its relatively isolated position at the crossroads of pollution from multiple sources.

The target plot of the model performance indicators delivers a combined matrix of the bias, standard deviation, and correlation of the model results with observations. The separate model performance criteria for each of these statistical metrics provide an in-depth analysis, highlighting the components that require improvement. Based on the case for Cyprus, we showed that the model is capable of reproducing the spatiotemporal distribution of ozone measured in the monitoring network that includes rural and urban areas, fulfilling the objectives set by the FAIRMODE evaluation methodology. Regarding nitrogen dioxide, there is a need for an improved, high-resolution representation of local emission fluxes to highlight the large urban-rural gradient in the observations. Particulate matter levels over the region are adequately simulated. However, the correlation between mean daily modeled and observed values needs further improvement. Overall, the model exhibits less variability in the pollutant concentration fields than the observations. The stringency of MQO related to PM2.5 must be further assessed.

The atmospheric aerosol distribution in the region is influenced by frequent mineral dust episodes, as well as other natural contributors such as sea salt and, especially during summer, black and organic carbon from accidental forest fires mostly upwind of the region (e.g., in southeastern Europe). For policy-related applications, the contribution of natural sources to mean daily and annual levels of pollutants must be well defined and the option to exclude the periods of episodes from the analysis must be provided, especially with regard to threshold exceedances. If this methodology is to be applied in forecasting applications, it must provide for solutions to separately assess events of episodic nature and distinguish between threshold exceedances from natural and anthropogenic sources.

Overall, the benchmarking evaluation methodology can provide comprehensive and detailed insights into the model performance related to regulated pollutants for scientific and policy applications. Since a large number of the objectives (and indicators) depend on the measurement uncertainty of the different pollutants, it is necessary to perform an assessment of this component for the region, due to its unique features regarding pollution, such as multiple source regions, chemical aging of atmospheric components during long-range transport, and regional climatic parameters.

## Electronic supplementary material

ESM 1 (PDF 248 kb)
