# Supplementary material for: Evaluation of EU air quality standards through modeling and the FAIRMODE benchmarking methodology
Source: Air Qual Atmos Health. 2018 Oct 24;12(1):73–86. doi: 10.1007/s11869-018-0631-z (PMC6327007; doi:10.1007/s11869-018-0631-z)
Supplement: Supplementary file 1 — (PDF 248 kb) [file 11869_2018_631_MOESM1_ESM.pdf]

ONLINE RESOURCE: Statistical metrics

Supplementary material for:

Evaluation of EU air quality standards through modelling and the FAIRMODE benchmarking methodology

Air Quality, Atmosphere & Health

Jonilda Kushta, Georgios K. Georgiou, Yiannis Proestos, Theodoros Christoudias, Philippe Thunis, Chrysanthos Savvides, Christos Papadopoulos and Jos Lelieveld

Corresponding author: Jonilda Kushta, j.kushta@cyi.ac.cy, Energy, Environment and Water Research Centre (EEWRC), The Cyprus Institute, Nicosia, 2121, Cyprus

CONTENTS:

Figure S1. Bar plots of daily maximum 8-hourly ozone a) annual mean, b) standard deviation, c) mean bias and d) correlation coefficient for the four rural background stations (Cavo Greco, Inia, Agia Marina and Troodos), three urban background stations (Larnaca, Limassol and Nicosia) and one industrial site (Zygi). Model results are indicated by red dots.

Figure S2. As in Figure S1 but for hourly NO2

Figure S3. As in Figure S1 but for daily PM2.5

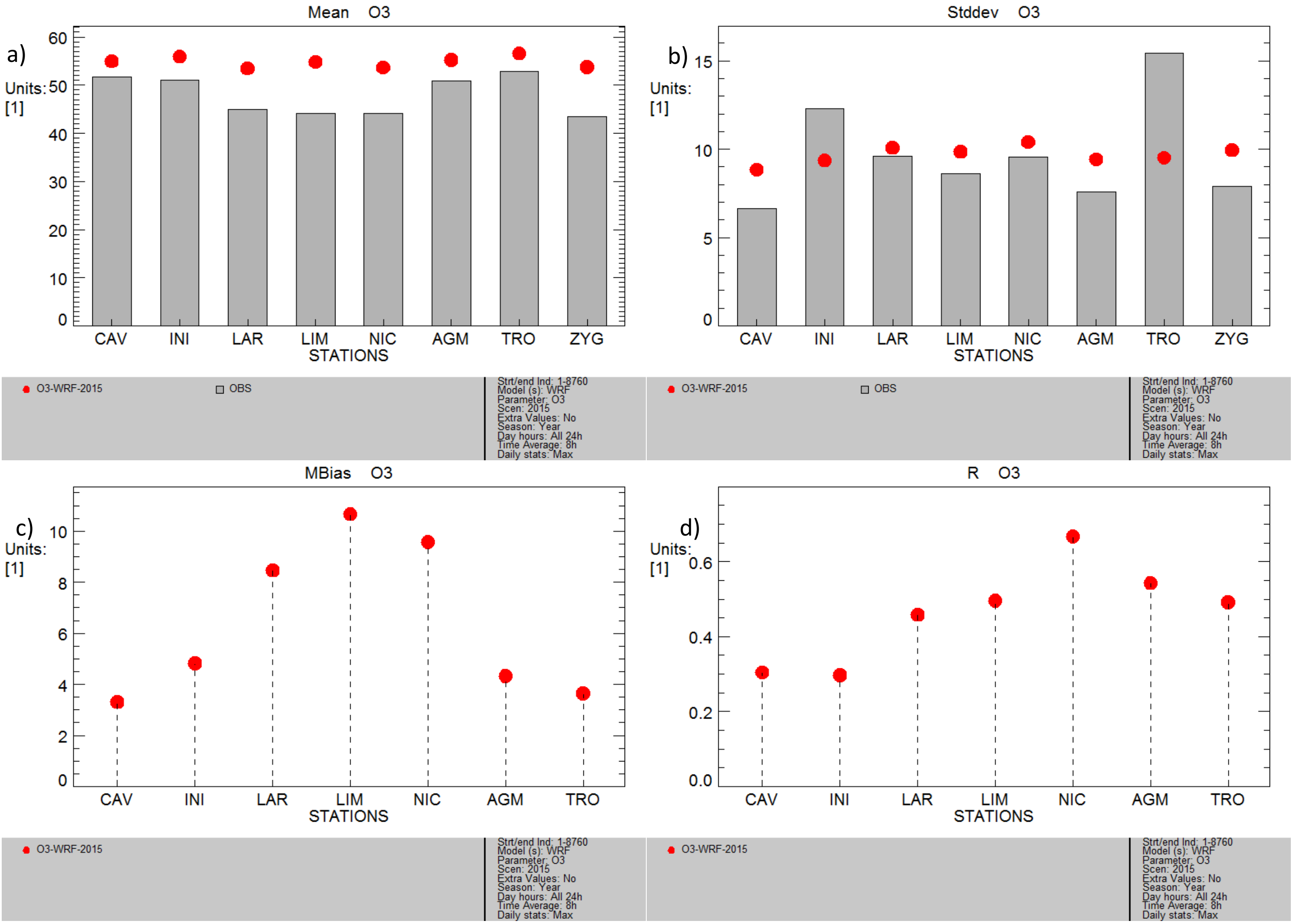

Figure S1. Bar plots of daily maximum 8-hourly ozone a) annual mean, b) standard deviation, c) mean bias and d) correlation coefficient for the four rural background stations (Cavo Greco, Inia, Agia Marina and Troodos), three urban background stations (Larnaca, Limassol and Nicosia) and one industrial site (Zygi). Model results are indicated by red dots.

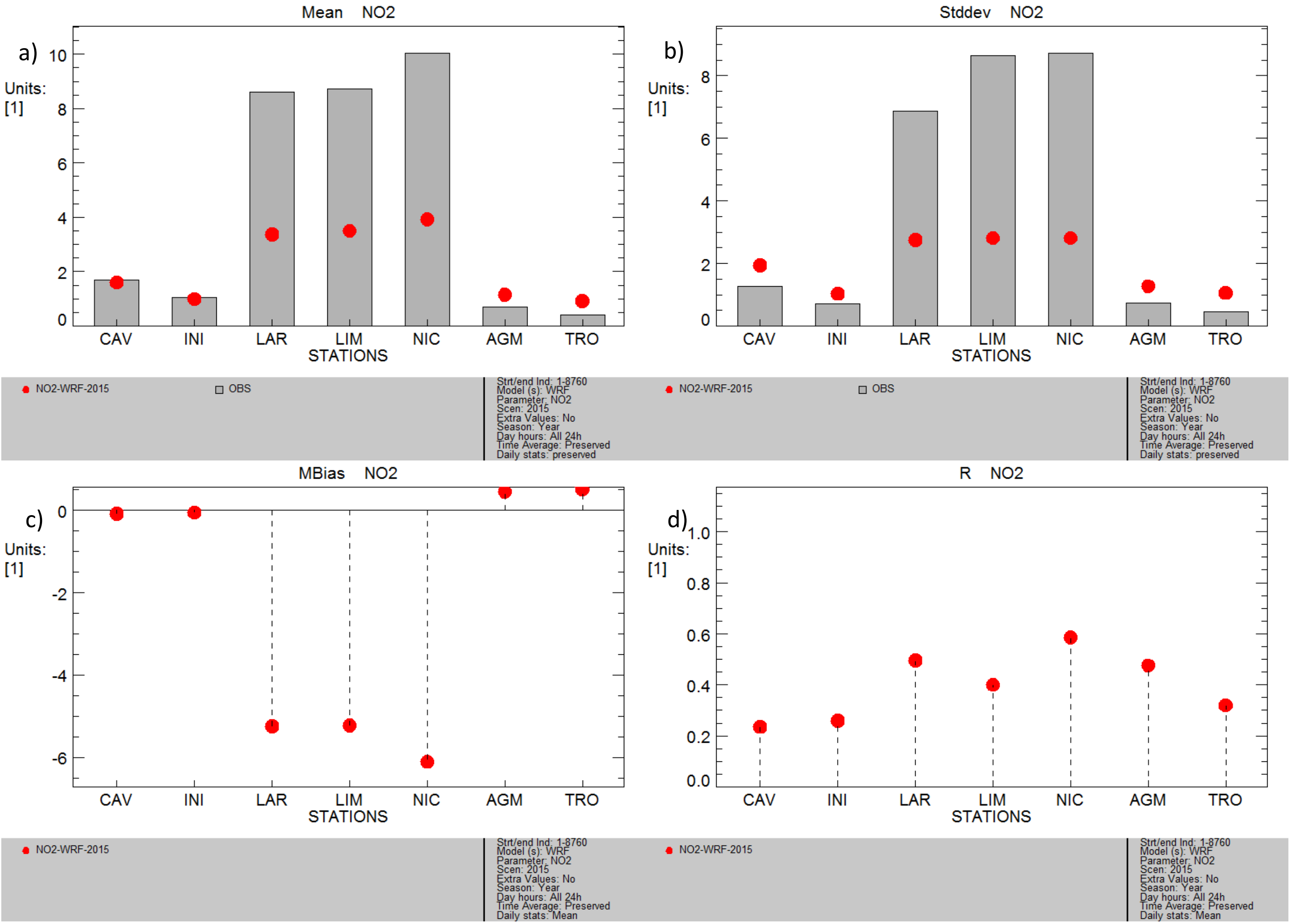

Figure S2. As in Figure S1 but for hourly NO_2

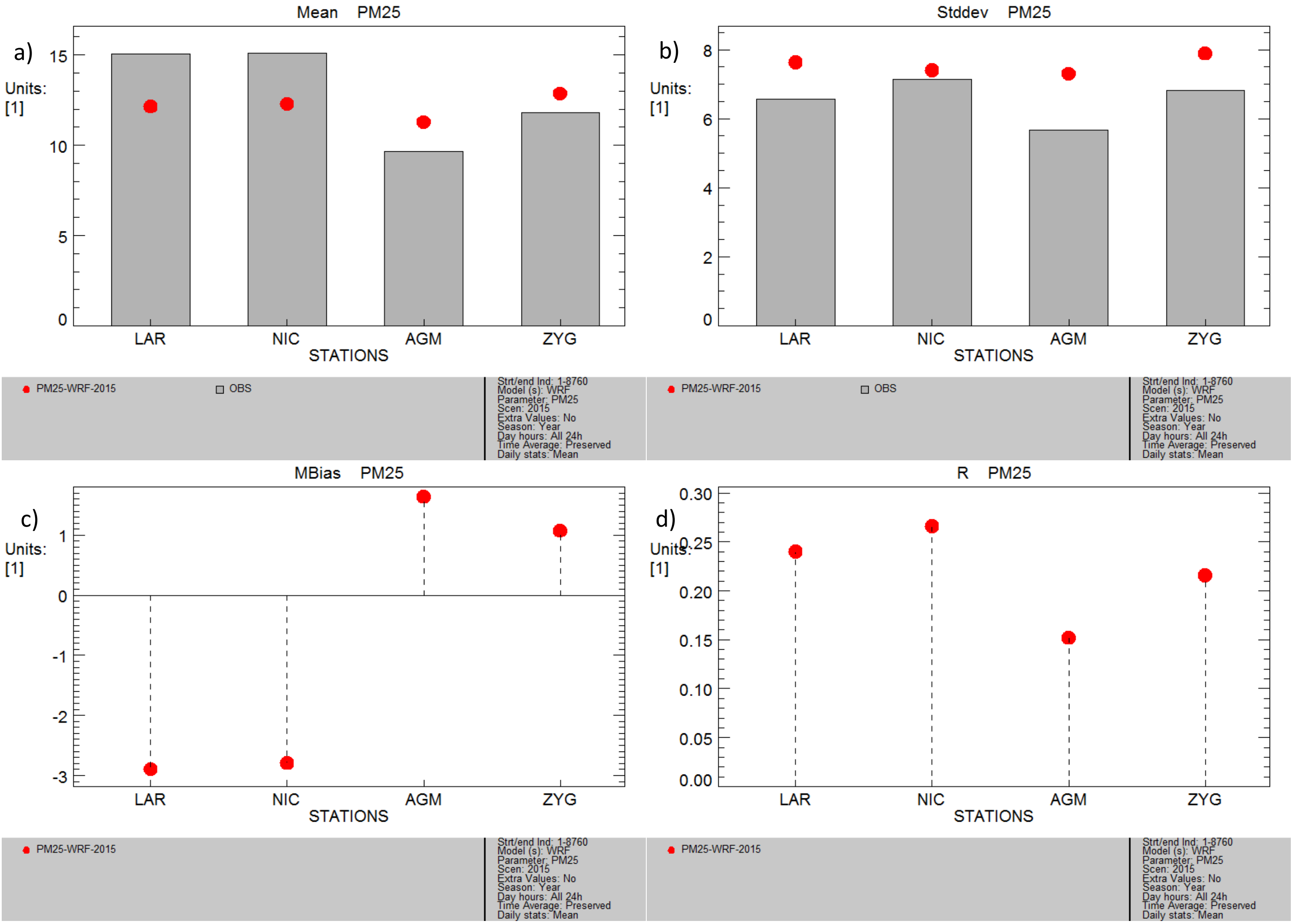

Figure S3. As in Figure S1 but for daily PM2.5
